# Experiences and perceptions of multidisciplinary paediatric teams of blended tube feeding in children

**DOI:** 10.1177/02601060231218049

**Published:** 2024-01-28

**Authors:** Orlaith Clancy, Siobhan McCormack, Meave Graham, Katie O’Sullivan, Annemarie E. Bennett

**Affiliations:** 1 Department of Clinical Medicine, Trinity Centre for Health Sciences, Trinity College Dublin, Dublin, Ireland; 2 Children's Health Ireland at Tallaght, Dublin, Ireland; 3 Children's Health Ireland at Temple Street, Dublin, Ireland

**Keywords:** Blended tube feeding, enteral feeding, paediatrics, paediatric multidisciplinary team, knowledge

## Abstract

**Background:** Blended tube feeding (BTF) is the administration of pureed whole foods via gastric feeding tubes. There is some evidence to suggest that BTF may have clinical and psychosocial benefits when compared to commercial formula, but further investigation of how BTF is understood and recommended by health professionals is needed. This study aims to investigate awareness and knowledge of BTF among multi-disciplinary paediatric staff in Ireland. **Methods:** A cross-sectional observational study was conducted among paediatric staff in Children's Health Ireland (CHI). The 16-item anonymous online survey gathered information on awareness of BTF, willingness to recommend BTF, confidence in BTF knowledge, and self-assessed competence in managing BTF. **Results:** Of the 207 responses, doctors (*n*68), nurses (*n*66), and dietitians (*n*32) provided 80.3% of responses. Two-thirds (*n*136, 66%) of the total group were aware of BTF. Of these, 68.1% had cared for a child on BTF and 70% (*n *= 63/90) were willing to recommend BTF. Three in five (*n *= 39/63, 61.9%) stated they were somewhat confident in their BTF knowledge and one in five (*n *= 12/56, 21.4%) were not yet competent in managing children on BTF. The most common reasons for recommending BTF were parental desire (*n*17, 39.5%) and commercial formula intolerance (*n*15, 34.9%). The most common barrier to recommending BTF was family logistics (*n*18, 41.9%). The most valuable sources of information on BTF for two-thirds (68.3%) of participants were other healthcare professionals (HCPs) and patients/caregivers. **Conclusion:** Healthcare settings should provide evidence-based training to HCPs on BTF to optimise the treatment and safety of children under their care.

## Introduction

Enteral nutrition (EN) is the provision of nutrition via a tube entering the gastrointestinal tract (NICE, 2017). A common route of EN is through a feeding tube inserted into a stoma that leads to the stomach, known as a gastrostomy (NICE, 2017). This is indicated when individuals with functional gastrointestinal tracts require long-term EN, are unable to obtain sufficient nutritional intake orally, or oral intake is unsafe (Gallagher et al., 2018; O'Connor et al., 2022; Samela et al., 2017; Serjeant and Tighe, 2022).

Commercial formula is the gold standard form of EN, offering a sterile and nutritionally complete method of feeding (Bennett et al., 2020; Carter et al., 2018; Chandrasekar et al., 2021). However, blended tube feeding (BTF), where pureed whole foods are administered enterally, has re-emerged in recent years (Bennett et al., 2020; Bobo, 2016; Carter et al., 2018) as a sole source of nutrition or as a supplement to commercial formula or oral intake (Coad et al., 2017; Pentiuk et al., 2011; Phillips, 2019). Despite the increasing prevalence of BTF (Folwarski et al., 2020; Mundi et al., 2017), there is a lack of consensus among healthcare professionals (HCPs) on how to balance patient/caregiver preferences with best practice guidelines for enteral feeding (Breaks et al., 2018; Köglmeier et al., 2023; O'Sullivan et al., 2019). While there is a growing body of evidence on the potential benefits of BTF, commercial formula is predominantly endorsed as the first line choice in EN policy, with policies citing the limited evidence base as a key impediment to endorsing BTF (Köglmeier et al., 2023). Contraindications to BTF may include patients who are immunosuppressed, have fine-bore gastrostomy tubes (<14fr), or are on fluid restrictions (Köglmeier et al., 2023; Schmitz et al., 2021); however, such contraindications are not absolute (Gallagher et al., 2018; Kernizan et al., 2020). Previously undiagnosed food allergies have been identified upon commencing BTF, cementing the necessity of HCP involvement to monitor the potential emergence of new BTF contraindications, especially in children without prior wholefood exposure (O'Connor et al., 2022).

As is the case with all forms of enteral feeding, ongoing monitoring of BTF by HCPs is pivotal to prevent the development of nutritional deficiencies and long-term adverse health outcomes (Chandrasekar et al., 2021; Schmitz et al., 2021; Santos and Morais, 2010), such as those seen in a case study of a child on BTF who developed scurvy due to low vitamin C provision (O’Hara, 2015). Due to the nature of their preparation, variability in the nutritional composition and completeness of blended tube feeds can occur (Ojo et al., 2020). Further, one of the most cited reasons for HCP reluctance towards BTF is the risk of microbial contamination due to improper food handling (Johnson et al., 2019; Milton et al., 2020), despite the presence of non-pathogenic bacteria being potentially beneficial (Bennett et al., 2020). No published study has linked increased infection rates with BTF, and safe food handling practices are effective in reducing bacterial loading (Milton et al., 2020). When carefully planned, BTF is generally well-tolerated and provides additional phytonutrients, prebiotics, and fibre, and can improve nutritional status and gastrointestinal symptoms (Gallagher et al., 2018; Hron et al., 2019; Martin and Gardner, 2017; Samela et al., 2017).

Feeding is an intimate interaction between caregivers and children. The desire to improve psychosocial experiences and quality of life at home is a common reason provided by caregivers for selecting BTF (Chandrasekar et al., 2021; Johnson et al., 2018; Soscia et al., 2021). Increased caregiver empowerment via the provision of blended whole foods reduces the medicalised approach to feeding and can enable caregivers to regain an aspect of parenting and family meals that may have been absent or reduced with commercial formula feeding (Edwards et al., 2016; Johnson et al., 2018; Pentiuk et al., 2011; Soscia et al., 2021; Trollip et al., 2020). However, caregivers may discontinue BTF due to time constraints and challenging preparation times (Trollip et al., 2020; Pentiuk et al., 2011). Difficulty with travel, equipment, storage, and fear of tube occlusions may contribute to discontinuation, despite the potential benefits of, and satisfaction with, BTF (Soscia et al., 2021; Trollip et al., 2020). Additionally, environments outside the home, such as schools, may not have the resources needed to support BTF (Shovlin et al., 2020). Despite these possible challenges to the feasibility of BTF, Pentiuk et al. reported that in a study of 33 children on BTF, parental satisfaction with BTF was high, with only one family discontinuing the feeding method on the grounds of inconvenience (Pentiuk et al., 2011). HCPs in a position to provide guidance on feeding should account for these types of factors to optimise caregiver preparedness and increase the feasibility of BTF as a sustainable method of feeding for as long as it is recommended.

Limited evidence exists on HCP knowledge and perceptions of BTF, with dietitians’ experience of BTF at the forefront of studies (Armstrong et al., 2017; Johnson et al., 2015; O'Sullivan et al., 2019). Dietitians have important roles in facilitating and monitoring BTF, but a reactive rather than proactive approach to BTF is common, and recommendations are often made secondary to intolerance to commercial formula or caregiver requests (Köglmeier et al., 2023; O'Sullivan et al., 2019). Concerns of nutritional inadequacy and tube occlusions (Armstrong et al., 2017), when combined with knowledge deficits, may contribute to resistance among HCPs towards BTF (Johnson et al., 2015).

Studies on this topic highlight that formal education and guideline awareness is lacking. This can inhibit HCPs from making evidenced-based recommendations on BTF. For example, in a study by Kariya et al. (Kariya et al., 2019), only a quarter of dietitians were confident in supporting families regarding BTF, and most respondents felt they were not knowledgeable or could not educate caregivers regarding BTF (Kariya et al., 2019). This can leave families unsupported and result in information being sought from sources that are not evidence-based (Johnson et al., 2018; Trollip et al., 2020). This is concerning when the paediatric population's evolving nutritional needs increase the likelihood that adverse nutritional outcomes may occur without appropriate oversight.

Given the increasing popularity of BTF, a patient-centred approach is needed from HCPs to ensure they are a key source of information for children and their families. This study aims to explore knowledge and experiences of BTF among paediatric staff by examining their perceptions, awareness, and desired sources of knowledge.

## Methods

This study was granted ethical approval from the Children's Health Ireland (CHI) Research Ethics Committee (REC-048-21). A cross-sectional observational study was conducted via an online survey.

Persons working across the CHI sites of Tallaght, Crumlin, and Temple Street were eligible to participate. Multidisciplinary paediatric clinical staff with the professional titles of doctor (all levels), nurse or advanced nurse practitioner (ANP), healthcare assistant, and AHPs were eligible if they worked in emergency departments, inpatient medical or surgical wards, intensive care units, outpatient departments or day wards. Eligible AHPs included dietitians, speech and language therapists, physiotherapists, occupational therapists, psychologists, and medical social workers.

Participants were invited to self-complete a 16-item online survey (Supplementary material) in March 2022. The survey was hosted on Qualtrics©. A participant information leaflet accompanied the survey. Written informed consent was provided at the outset of the survey. The survey was informed by two studies (Armstrong et al., 2017; O'Sullivan et al., 2019), and contained questions adapted from these studies with the authors’ permission. The survey was not validated. Four closed-ended questions gathered information on demographics and seven closed-ended questions explored participants’ experience and perceptions of BTF. Participants who had cared for a child on BTF were eligible to complete five closed-ended questions on whether they would recommend BTF; confidence in their knowledge; competence; reasons for recommending BTF in practice; and, barriers to recommending BTF. Finally, five closed-ended questions explored sources of information used to facilitate professional development on BTF and what further information participants desired.

Estimated time for survey completion was 5–7 min. A pilot study involving 9 paediatric dietitians, nurses, and doctors across the study sites provided feedback on the survey prior to dissemination, to ensure survey functionality. Following the receipt of feedback, no questions were altered, but minor adjustments to phrasing, layout, and the addition of potential answers to questions were made. The survey link was disseminated via email to eligible participant groups across CHI. Posters with the survey link and a QR code were placed in high-footfall areas across CHI, i.e., areas that hospital staff regularly travel through, such as outpatient department areas for staff, staff canteens/breakrooms, and receptions. Physical survey stands were also established near receptions and canteens in CHI to optimise the response rate by recruiting staff in areas where they were more likely to have time to answer the survey.

Data analyses were conducted using the SPSS for macOS, version 28.0 (IBM Corporation, Armonk, New York). Categorical data were presented utilising counts and proportions. The association and differences between categorical variables were assessed using cross-tabulations, and the Chi-squared statistics test was used to assess statistical significance. Statistical significance was taken at *p *≤ 0.05.

## Results

### Participant demographics

Two-hundred-and-fifteen responses were recorded, with 207 eligible for analysis and a 92% completion rate. The number of eligible participants across three CHI hospitals (Crumlin, Tallaght, Temple Street) at the time of the survey was approximately 2595, giving an estimated response rate of 8.3%. The largest proportion (39.6%, *n*82) of participants were staff based in CHI at Tallaght, while a third (33.8%, *n*70) and 26.6% (*n*55) were based in CHI at Temple Street. As shown in Table 1, 80.3% (*n*166) of responses were provided by doctors (32.9%), nurses (31.9%) and dietitians (15.5%).

**Table 1.** Professional characteristics of 207 clinical staff across Children's Health Ireland.

Characteristic	*n*	%
Professional Title		
Doctor (All levels)	68	32.9
Nurse or ANP	66	31.9
Healthcare Assistant	13	6.3
Dietitian	32	15.5
Speech and Language Therapist	5	2.4
Physiotherapist	9	4.3
Occupational Therapist	3	1.4
Psychologist	7	3.4
Medical Social Worker	4	1.9
**Professional Experience (years)**		
0–5	67	32.4
5–10	54	26.1
10–15	18	8.7
15–20	23	11.1
>20	45	21.7

### Awareness, experience and attitudes

One-third (33.9%, *n*70/206) had not heard of BTF and 65.7% (*n*136/206) had heard of BTF. There was no statistically significant difference [*p *= 0.246] between BTF awareness among those with ≤10 years’ and those with >10 years’ professional experience. As shown in Table 2, 69.1% of doctors and 60.0% of nurses had heard of BTF, compared to 96.9% of dietitians.

**Table 2.** Awareness of blended tube feeding (BTF) among 206 paediatric health professionals.

	Yes - I have heard of BTF		No – I have not heard of BTF	
Professional Title	*n*	%	*n*	%
Doctor	47	69.1	21	30.9
Nurse or Advanced Nurse Practitioner	39	60.0	26	40.0
Healthcare Assistant	2	15.4	11	84.6
Dietitian	31	96.9	1	3.1
Speech and Language Therapist	5	100.0	0	-
Physiotherapist	5	55.6	4	44.4
Occupational Therapist	1	33.3	2	66.7
Psychologist	4	57.1	3	42.9
Medical Social Worker	2	50.0	2	50.0

Of those who had BTF awareness (*n*136) (Table 3), two-thirds (68.1%, *n*92/135) had provided care to a child on BTF. Over two-thirds (70.0%, *n*63/90) of respondents with BTF experience indicated a degree of willingness to recommend BTF, while a quarter (27.8%, *n*25/90) felt they did not know enough to recommend BTF (Table 3). Most doctors (84.8%, *n*28/33) and nurses (66.7%, *n*14/21) would recommend on a case-by-case basis or stated that they did not know enough to recommend. Dietitians predominantly reported recommending BTF on a case-by-case basis (78.3%, *n*18/23).

**Table 3.** Health professionals’ experience of, and attitudes towards, blended tube feeding (BTF).

	n	%
**Experience managing a patient on BTF (*n*135)**		
Yes	92	68.1
No	43	31.9
**Willingness to recommend BTF (*n*90)**		
Do not know enough to recommend	25	27.8
Would not recommend	2	2.2
Recommend on a case-by-case basis	47	52.2
Might recommend	10	11.1
Would definitely recommend	6	6.7
**Confidence in knowledge of BTF (*n*63)**		
Not confident at all	6	9.5
Not very confident	9	14.3
Somewhat confident	39	61.9
Very confident	9	14.3
**Competence in ability to support BTF (*n*56)**		
Not yet competent on BTF	12	21.4
Knowledgeable on BTF	24	42.9
Confidently manage a patient on BTF	13	23.2
Have expertise designing diet plans to meet nutritional needs	6	8.9
Have experience administering BTF	2	3.6

Three in five (61.9%, *n*39/63) respondents with BTF experience and who were willing to recommend BTF, felt somewhat confident in their knowledge (Table 3). Most doctors (76.0%, *n*19/25) and dietitians (87.0%, *n*20/23) who were willing to recommend BTF to some degree, felt somewhat or very confident in their knowledge.

While a substantial proportion (42.9%, *n*24/57) of respondents with experience and who were willing to recommend BTF self-assessed as knowledgeable, 21.4% (*n*12) self-assessed as not yet competent in their abilities to manage BTF (Table 3). Doctors (52.2%, *n*12/23) and nurses (66.7%, *n*6/9) reported greater knowledge than competence in the practical management of BTF. However, dietitians (59.1%, *n*13/22) reported greater competence in the management of BTF and in designing feeding plans.

### Perceptions of barriers and benefits

The most common reasons to recommend BTF were parental desire (39.5%, *n*17/43), symptoms of commercial formula intolerance (34.9%, *n*15/43), and psychosocial considerations (23.3%, *n*10/43). Doctors cited parental desire (60.0%, *n*9/15) as a key reason to recommend BTF, whereas dietitians based their recommendations on current symptoms (*n*11/20, 55%).

The most common barriers to recommending BTF were concerns around family logistics (41.9%, *n*18/43) and risk of nutritional inadequacy (20.9%, *n*9/43). Doctors commonly cited family logistics (53.3%, *n*8/15), dietitians cited nutritional inadequacy and family logistics equally (40.0%, *n*8/20 each), and nurses cited tube occlusions (66.7%, *n*4/6) as the biggest barrier.

### Previous information received

Two-thirds (68.3%, *n*43/63) of participants had received information on BTF and almost a third (31.7%, *n*20/63) had not. Those who reported competence in their ability to manage patients on BTF had all received previous information on BTF. Similarly, when they had previously received information on BTF, participants were more likely to report being knowledgeable (65.2%, *n*15/23), able to confidently manage BTF (84.6%, *n*11/13), or being somewhat (76.3%, *n*29/38) or very (77.8%, *n*7/9) confident with their BTF knowledge. The most valued sources of information on BTF were speaking with other health professionals (90.7%, *n*39/43) and learning from patients/caregivers (51.2%, *n*22/43).

### Information desired

Most (92.4%, *n*121) respondents expressed an interest in receiving further information on BTF (Table 4). The information most desired was how to monitor children on BTF (25.6%, *n*86), evidence on BTF (24.7%, *n*83), and how to plan a nutritionally adequate BTF diet (22.6%, *n*76). The most commonly desired methods of information delivery were written information (26.3%), face-to-face learning (25.9%), and pre-recorded online lectures (25.5%) (Table 4).

**Table 4.** Desired information and methods of delivering information on blended tube feeding.

	n	%
**Further information desired (*n131)***		
Yes	121	92.4
No	10	7.6
Information type desired (*n*112)*		
How to monitor children on BTF	86	25.6
Evidence behind the diet	83	24.7
How to plan a nutritionally adequate diet	76	22.6
Case reports and patient experiences	60	17.9
How to store and administer the BTF	31	9.2
Desired methods of delivery of structured education of BTF (*n*112)*		
Written Information resources	59	26.3
Face-to-face learning/workshops	58	25.9
Pre-recorded online lectures	57	25.5
Live online learning	34	15.2
Self-directed learning	16	7.1

*Multiple responses per respondent permitted.

## Discussion

This is the first study exploring multidisciplinary paediatric clinical staff perceptions of BTF in Europe. Two-thirds of participants in this study had heard of BTF, with 69.1% of doctors and 60% of nurses being aware of BTF, compared to 97% of dietitians. Awareness of BTF among HCPs appears greater in the present study compared to a U.S. study of comparable sample size (Eustace et al., 2021), where 50.4% of respondents were familiar with BTF, to include 55.1% of all physicians and 46.6% of all nurses (Eustace et al., 2021). However, the authors explored physician, physician assistants, and nurses’ perspectives of BTF in adult and paediatric populations, compared to the broader range of professionals included in this study. While awareness is greater generally and within distinct professions in the present study, BTF awareness differs by area of practice. In a U.S. study (Eustace et al., 2021), only 40% of respondents indicated paediatrics as their dominant area of practice. As such, future studies are needed to explore whether HCPs in adult or paediatric settings have greater BTF awareness, to elucidate differences in education needs by setting. The lack of awareness surrounding BTF may also reflect the emphasis among respondents on the need for educational resources on this feeding modality (Eustace et al., 2021). A lack of awareness among HCPs may result in parents engaging in unsafe feeding practices if not advised appropriately (Carter et al., 2018; Chandrasekar et al., 2021; Serjeant and Tighe, 2022). This is concerning considering many children on BTF have medically and nutritionally complex needs, increasing the likelihood of adverse clinical outcomes occurring due to a lack of knowledgeable oversight (Carter et al., 2018).

Confidence in this study referred to confidence in the accuracy of one's BTF knowledge. Of those with professional experience of BTF, a quarter felt they did not know enough to recommend BTF and over half would recommend on a case-by-case basis. However, the majority still only felt somewhat confident in their BTF knowledge. Eustace et al. (Eustace et al., 2021) reported in their study that respondents expressed a high willingness to support BTF despite the majority having no opinion on recommending BTF and low confidence in BTF knowledge. While there was a willingness to recommend, a lack of confidence was identified in the current study, even after managing a child on BTF, likely highlights the need for formal BTF guidance for HCPs.

In this study, dietitians were the most confident in their BTF knowledge. Over three-quarters recommended BTF on case-by-case basis; a finding that appears to have progressed since a 2019 study (O'Sullivan et al., 2019) in Ireland, where less than half of paediatric dietitians expressed a willingness to support BTF if requested. Moreover, a third of dietitians stated they might recommend BTF and over a quarter had no opinion (O'Sullivan et al., 2019), compared to a Scottish survey reporting in 2017 that 44.2% of dietitians would generally not recommend BTF and 14.3% would advise against it (Armstrong et al., 2017). This highlights that willingness to recommend BTF amongst dietitians may be increasing. This may be due to formal guidance emerging since the publication of these papers, such as the British Dietetic Association's toolkit on BTF management.

It is evident that the increased provision of formal supports for HCPs is warranted. Of the participants in this study who had BTF experience, almost a third had not received information on BTF. Of those who had received information, speaking with health professionals and learning from children and caregivers were their most valuable sources of knowledge. These informal knowledge sources are echoed in literature, where Kariya et al. reported that 27% of dietitians received no BTF education of any kind. Of those who had previous education, self-directed learning, learning from colleagues, and learning from patients were the top sources of education (Kariya et al., 2019). In contrast, a study on dietitians in Ireland reported that their most cited sources of information were international guidelines and journal articles (O'Sullivan et al., 2019). It has been noted in literature that HCPs, outside of dietitians, rely on dietitians as their main source of BTF information (Eustace et al., 2021). This expectation of expertise may drive dietitians towards formal sources of information on BTF. Notably, Eustace et al. identified that almost all respondents in their study had no previous professional guidance on BTF (Eustace et al., 2021). The lack of formal education provision results in HCPs seeking information from sources that may not have an appropriate evidence base, possibly contributing to the hesitancy of HCPs to recommend BTF.

Overall, 92.4% of respondents in this study expressed an interest in learning more on BTF. Similarly, in a 2017 UK study of 188 dietitians, 90% of respondents wanted further information on BTF (Cantwell and Ellahi, 2017). Regardless of prior experience, the present study suggests that HCPs are open to learn about BTF via a range of methods of information delivery (e.g., face-to-face learning and pre-recorded lectures). Formal and further education may prove effective in enhancing confidence, competence, and willingness to recommend BTF where appropriate.

The strengths and limitations of this study must be considered. Strategies to improve the response rate included convenience sampling, use of physical stands in high-footfall areas in the hospital sites, and snowball sampling. Limitations include the observational study design, which prevents causal inferences from being drawn. The survey was not validated, but no validated survey exploring paediatric HCPs' perspectives on BTF currently exists in literature. Whilst the three hospitals used to recruit participants represent the largest centre for paediatric care in the Republic of Ireland, the sample is not nationally representative. Finally, the survey response rate was comparable to some similar research conducted utilising online surveys in this area (Johnson et al., 2015; O'Sullivan et al., 2019), but was below average when compared to more recent similar studies conducted (Eustace et al., 2021; Kariya et al., 2019). Further research is needed on HCPs perceptions of BTF, with qualitative research methodologies being especially valuable. The development of a standardised survey tool would also be beneficial to compare BTF awareness and knowledge nationally and internationally.

BTF remains a patient- and demand-led service (Köglmeier et al., 2023). This study highlights that HCPs are at risk of providing suboptimal care to patients on BTF due to a lack of awareness and confidence and limited formal training. As HCPs have distinct roles when managing BTF, the provision of tailored education specific to relevant professions would be ideal, but given the current low baseline of education provision, even general formal education for all professions is prudent to ensure that a minimum standard of safe care for vulnerable children on BTF is met.

## Supplemental Material

sj-pdf-1-nah-10.1177_02601060231218049 - Supplemental material for Experiences and perceptions of multidisciplinary paediatric teams of blended tube feeding in children Supplemental material, sj-pdf-1-nah-10.1177_02601060231218049 for Experiences and perceptions of multidisciplinary paediatric teams of blended tube feeding in children by Orlaith Clancy, Siobhan McCormack, Meave Graham, Katie O’Sullivan and Annemarie E. Bennett in Nutrition and Health
