# Supplementary material for: Experiences and perceptions of multidisciplinary paediatric teams of blended tube feeding in children
Source: Nutr Health. 2024 Jan 28;31(2):681–8. doi: 10.1177/02601060231218049 (PMC12174607; doi:10.1177/02601060231218049)
Supplement: sj-pdf-1-nah-10.1177_02601060231218049 - Supplemental material for Experiences and perceptions of multidisciplinary paediatric teams of blended tube feeding in children [file sj-pdf-1-nah-10.1177_02601060231218049.pdf]

Blended Tube Feeding Staff Survey and Participant Information Leaflet

Children's Health Ireland

SPIRE

Severe neurological imPairment in IREland

Trinity College Dublin
The University of Dublin

Participant Information– Staff survey

"Multi-disciplinary paediatric teams' experiences and perceptions of blended tube feeding in children"

You are being invited to take part in a research survey on blended tube feeding that is being carried out across CHI at Tallaght, CHI at Temple Street and CHI at Crumlin, led by the Department of Child Development and Neurodisability at CHI at Tallaght and the Department of Dietetics at Trinity College Dublin and TU Dublin.

Before you decide whether or not you wish to take part please read the information provided below:

Part 1 – The Study

Why is this study being done?

We are doing this study in an effort to explore the experience of paediatric clinical staff of CHI regarding a type of tube feeding method called the "blended diet". Blended tube feeding involves whole or real table foods including vegetables, fruit, meat, legumes and whole grains that have been blended, pureed or liquidised and given via a feeding tube. Children on tube feeds access services throughout CHI including ED, outpatients, inpatient wards, ICU, general paediatrics, general surgery, neurodisability, neurology, gastroenterology, cardiology, haem/oncology, neonatology, respiratory and metabolic. Most children are on commercial formula feeds as standard, but there has been a recent increase in families who wish to enterally feed their children via blended tube feeds. There is a lack of high-quality evidence from clinical trials to support blended tube feeds over commercial feeds, but many studies describe parent reported benefits in terms of improving gastrointestinal symptoms and parental satisfaction. This study will characterise staff awareness of this method, experience of it, perceptions around the diet and supporting families to use it, along with helping to understand what information staff would like on this method and how they might like that

education to be delivered.

Why have I been invited to take part?

You are invited to take part in this survey because you are a staff member of CHI in the following areas: Emergency department, inpatient medical or surgical wards, ICU, OPD, day wards or: you work in the following roles: Doctor, Nurse or ANP, Healthcare assistant, Dietitian, Speech and Language Therapist, Physiotherapist, Occupational Therapist, Psychologist, Medical Social Worker. We would like to capture all specialties with the exception of: radiology, lab, pharmacy, orthopaedic surgery, neuro-surgery, plastic surgery and ENT.

Do I have to take part? Can I withdraw?

No, this is entirely voluntary, you do not have to participate at all. As the survey is anonymous it will not be possible to withdraw after submission.

What happens if I change my mind?

You can change your mind by not submitting the survey.

How will the study be carried out?

This survey will take place from start of March 2022 for 4 weeks. We hope to enrol 200-400 staff members. The more staff that take part the more helpful our results will be.

Are there any benefits to taking part in this research?

Taking part in this study will not directly benefit you but the information that is collected will aim to enable us to plan training and education for staff and improve the services we provide to the children and families we look after.

Are there any risks to me or others if I take part?

No harm is anticipated for you from participating in this study.

What will happen to me if I decide to take part?

You will be asked to read this leaflet, and complete the survey. The survey will take about 5 minutes.

What will happen to my data?

The data from this survey is anonymous and we will not be collecting personal data.

Will I be told the outcome of the study?

The overall outcomes of the study will be presented at the hospital research days, national and international conferences and may be published in medical journals.

Part 2 – Data Protection

What information about me (personal data) will be used as part of this study?

No identifying data (name, date of birth, address, employee or phone number) will be required.

Part 3 – Costs, Funding and Approval

Has this study been approved by a research ethics committee?

The study has obtained and been granted ethical approval from the ethics committee of CHI on the 19th of January 2022.

Who is organising and funding this study? Will the results be used for commercial purposes?

This research is undertaken by the Department of Child Development and Neurodisability, CHI at Tallaght, Severe neurological imPairment in IREland (SPIRE) study group along with co-investigators from CHI at Temple St and CHI at Crumlin and final year dietetics student Ms Órlaith Clancy under the academic supervision of Dr Annemarie Bennett of Trinity College Dublin. The results will not be used for commercial purposes.

Is there any payment for taking part?

No, we are not paying participants to take part in the survey.

Part 4 – Future Research

Will my personal data be used in future studies?

No personal data is collected as part of this survey.

Part 5 – Further Information

Who should I contact for information or complaints?

You can get more information on this study by contacting: Department of Child Development and Neurodisability, Children's Health Ireland at Tallaght, Tallaght University Hospital, Dublin 24. Email: spirestudy@tuh.ie

Thank you for taking the time to read this information sheet and for considering taking part in this study.

End of Block 1

Start of Block 2

Section 1. Questions on Demographics

Q1 In which of the following sites are you principally based?

- ○ CHI at Tallaght
- ○ CHI at Temple Street
- ○ CHI at Crumlin
- ○ CHI at Connolly
- ○ None of the above

Skip To: End of Survey if: In which of the following sites are you principally based? = None of the above

Q2 What is your role in this site?

- ◯ Doctor
- ◯ Nurse or ANP
- ◯ Healthcare Assistant
- ◯ Dietitian
- ◯ Speech and Language Therapist
- ◯ Physiotherapist
- ◯ Occupational Therapist
- ◯ Psychologist
- ◯ Medical Social Worker
- ◯ None of the above

Skip To: End of Survey if What is your role in this site? = None of the above

Q3 Where is your main area(s) of work in this site?

- ◯ Emergency Department
- ◯ Inpatient medical ward
- ◯ Inpatient surgical ward
- ◯ ICU
- ◯ OPD
- ◯ Day Ward
- ◯ None of the above

Q4 How many years of experience do you have in this line of work?

- 0-5
- 5-10
- 10-15
- 15-20
- >20

End of Block 2

Start of Block 3

Section 2. Questions on MDT Current Perceptions and Understanding of BTF

Q5 Have you ever heard of blended tube feeding previously?

- Yes
- No

Skip To: End of Survey if Have you ever heard of blended tube feeding previously? = No

Q6 Have you looked after a child on a blended tube feed?

- Yes
- No

Skip To: End of Block if Have you looked after a child on a blended tube feed? = No

Q7 Would you recommend the use of blended tube feeding to parents?
Select the answer that best reflects your views:

○ I do not feel that I know enough to recommend/advise the use of blended tube feeding to parents

○ I would definitely recommend the use of blended tube feeding

○ I would recommend the use of blended tube feeding on a case-by-case basis

○ I might recommend the use of blended tube feeding

○ I would not recommend blended tube feeding

○ I would actively advise parents against blended tube feeding

Skip To: End of Block if Would you recommend the use of blended tube feeding to parents?   Select the answer that best ref... = I do not feel that I know enough to recommend/advise the use of blended tube feeding to parents

Skip To: Q12 if Would you recommend the use of blended tube feeding to parents?   Select the answer that best ref... = I would not recommend blended tube feeding

Skip To: Q12 If Would you recommend the use of blended tube feeding to parents?   Select the answer that best ref... = I would actively advise parents against blended tube feeding

---

Q8 How confident are you in your knowledge to support parents using blended tube feeds?
Select the answer that best reflects your views:

○ I would feel not confident at all in my knowledge of blended tube feeds

○ I would feel not very confident in my knowledge of blended tube feeds

○ I would feel somewhat confident in my knowledge of blended tube feeds

○ I would feel very confident in my knowledge of blended tube feeds

Skip To: Q12 if How confident are you in your knowledge to support parents using blended tube feeds?   Select the... = I would feel not confident at all in my knowledge of blended tube feeds

Q9 How competent do you feel in your ability to support parents who are using blended tube feeds? Select the answer that best reflects your views:

- ○ I am not yet competent on blended tube feeds
- ○ I am knowledgeable regarding blended tube feeds
- ○ I can confidently manage a patient who has chosen blended tube feeds
- ○ I have the expertise to design blended tube feeding diet plans to meet nutritional goals of patients
- ○ I have the experience of administering blended tube feeds to patients

Skip To: Q12 if How competent do you feel in your ability to support parents who are using blended tube feeds? Selected... = I am not yet competent on blended tube feeds

Q10 What would be the primary reason for you to recommend blended tube feeding? Select the answer that best reflects your views:

- ○ Parental desire to try blended tube feeding
- ○ Current symptoms of tube feed intolerance on commercial formula
- ○ Psychosocial considerations for patients and their family (i.e. improved mealtime engagement)
- ○ Other ____

Q11 What would be the main barrier when considering recommending blended tube feeding? Select the answer that best reflects your views:

- ○ There is no factor that would act as barrier when recommending blended tube feeding
- ○ Risk of nutritional inadequacy
- ○ Risk of food borne microbial infection
- ○ Concerns surrounding familial logistics to support blended tube feeding (?)
- ○ Risk of tube occlusions
- ○ Inadequate patient-friendly resources and guidance to support use
- ○ Unsuitable patient type for this method of feeding
- ○ Other ____

(?) E.g. access to cooking equipment, access to safe food storage and preparation, needs of any other children in the household, socioeconomic status of household

Section 3. Questions on BTF Information.

Q12 Have you ever received any previous information on blended tube feeds?

- ○ Yes
- ○ No

Skip To: End of Block if Have you ever received any previous information on blended tube feeds? = No

Q13 What sources of information on managing blended tube feeds have you found most valuable to date?
Select two sources that you found most valuable:

- ○ Speaking with other health professionals (including dietitians)
- ○ Medical journals
- ○ Internet
- ○ Learning from patients
- ○ Workplace education sessions
- ○ Conferences
- ○ Social Media
- ○ International guidelines

End of Block 3

Start of Block 4

Q14 Would you like more information on blended tube feeds?

○ Yes

○ No

Skip To: End of Block if Would you like more information on blended tube feeds? = No

Q15 What information would you like on blended tube feeds?

Rank the top three answers that would be most important to you:

(Click on the options to the left and drag across to the relevant box on the right)

Choices Available	Choice 1	Choice 2	Choice 3
1. Information on the evidence behind this diet			
2.Information on how to plan the diet to ensure nutritional adequacy			
3.Information on storing and administering of the blended feed			
4.Case reports and experiences from children on blended tube feeds			

5.Information on how to monitor children on blended tube feeds

Q16 How would you prefer to receive structured education on blended tube feeding? Select two answers from the list below:

- ○ Pre-recorded online learning
- ○ Live online learning
- ○ Face to face learning/workshops
- ○ Written information resources
- ○ Self-directed learning

End of Block 4

Start of Block 5

Any further comments?

____

____

____

____

____

End of Block 5

End of Survey

We thank you for your time spent taking this survey.

Your response has been recorded.
